# Immunomodulatory Effects of Dietary Phosphorus and Calcium in Two Strains of Laying Hens

**DOI:** 10.3390/ani11010129

**Published:** 2021-01-08

**Authors:** Tanja Hofmann, Sonja Schmucker, Vera Sommerfeld, Korinna Huber, Markus Rodehutscord, Volker Stefanski

**Affiliations:** Institute of Animal Science, University of Hohenheim, 70599 Stuttgart, Germany; sonja.schmucker@uni-hohenheim.de (S.S.); v.sommerfeld@uni-hohenheim.de (V.S.); korinna.huber@uni-hohenheim.de (K.H.); markus.rodehutscord@uni-hohenheim.de (M.R.)

**Keywords:** laying hen, phosphorus, calcium, immune system, health

## Abstract

**Simple Summary:**

Phosphorus and calcium are essential nutrients for body functions including the immune system and are generally supplemented to poultry diets. Phosphorus is also present in plant feedstuffs, bound as phytate, which can be used by enzymatic hydrolyzation in the chicken. A reduction of dietary mineral phosphorus might consequently be conceivable, without negatively influencing the immune system. The high concentration of calcium in diets for laying hens that is needed for eggshell formation may inhibit phytate degrading enzymes, and thus, decrease phosphorus availability for the hen. Both phytate degradation and several immune parameters are known to be strain-specific, making an interaction of the genetic background and the dietary phosphorus and calcium supply with the immune system likely. The aim of the study was to evaluate the impact of reduced concentrations of dietary phosphorus and calcium on the peripheral and gut-associated immune system in two laying hen strains. Reduced mineral phosphorus enhanced, while reduced calcium reduced several immune parameters. The two strains showed differences in many immune parameters, but only the impact of dietary phosphorus was influenced by the genetic background. These results suggest that dietary phosphorus and calcium supply may strain-specifically influence immune defense and protection against infection in chicken.

**Abstract:**

Insufficient nutrient supply can impair the immune system, which is important for animal health and welfare. Since chicken can partly hydrolyze phytate, which is the primary phosphorus storage in plant seeds, a reduction of mineral phosphorus in the diets could be an option for more sustainable egg production. Laying hens require high concentrations of calcium that might inhibit the function of endogenous enzymes for phytate hydrolyzation. The objective of this study was to characterize the impact of standard and reduced dietary phosphorus and calcium concentrations on the number and functionality of immune cells in the peripheral and gut-associated immune system in a white and brown laying hen strain. Reduced mineral phosphorus enhanced several immune parameters such as B cells in blood and IgA concentrations in bile in both strains, and peripheral monocytes and γδ T cells in cecal tonsils in brown hens. Reduced calcium levels resulted in lower numbers of T cells in blood and cecal tonsils in both strains, suggesting negative effects on adaptive immunity. Differences between the two strains were found in almost all immune parameters. Results suggest a potentially beneficial effect of reduced dietary mineral phosphorus on the immune system that is dependent on the genetic background.

## 1. Introduction

Phosphorus (P) and calcium (Ca) are essential nutrients for bone development and various biochemical pathways in all organisms [1]. In plant-based diets, P is mainly present in the form of phytic acid (*myo*-inositol 1,2,3,4,5,6-hexakis (dihydrogen phosphate); InsP_6_) and its salts (phytate). Nonruminant animals have long been assumed to lack sufficient endogenous mucosal phytase and phosphatase to hydrolyze InsP_6_ to lower *myo*-inositol phosphates (InsP_x_) and *myo*-inositol, thus making phytate-P biologically available. Therefore, diets for nonruminant animals are usually supplemented with inorganic P or exogenous phytase, or both, to cover the P requirements. The supplementation of phytase is very cost-intensive and an increase in the concentration of P in manure may have a negative impact on the environment [2]. However, recent studies demonstrated the high potential of poultry to hydrolyze phytate by mucosal enzymes and gut bacteria [3,4,5], when fed diets low in mineral P and Ca. In general, P concentration is closely related to Ca concentration as their metabolism and absorption rate are interrelated [6,7]. For laying hens, the availability of both nutrients is most crucial during the laying period [8], with a high requirement for dietary Ca due to eggshell formation and less requirement for P [9]. This results in different conditions for phytate degradation compared to broiler chickens, suggesting more interactions with P and Ca utilization, phytate hydrolysis, and *myo*-inositol release.

Many authors conclude that mineral P concentration in feed of laying hens may be too high and thus could be reduced [2,9,10]. However, the impact of low dietary P and Ca on the peripheral and gut-associated immune system has not yet been investigated in laying hens. Prerequisite for animal health is a fully functional immune system. With the ongoing intensification of the poultry industry and the parallel demand for reduced use of antibiotics, it becomes even more important to maintain or improve poultry health. Several factors that may modulate the avian immune system are known, including nutrition [11]. The effects of dietary P on the immune system and intestinal microbiota in livestock species such as swine, cattle, and broiler chickens were recently reviewed, suggesting an overall positive impact of dietary available P and phytase addition on the adaptive immune response, although results remained inconsistent [12]. In another recent study with broilers, higher P availability due to higher nonphytate-P or the addition of exogenous phytase was also shown to improve antibody titers against infectious bronchitis virus and cutaneous hypersensitivity [13], suggesting an enhanced adaptive cellular and humoral immune response. Moreover, laying hens showed higher IFN-γ concentration in blood when fed higher levels of nonphytate-P, also indicating enhanced immune function [14].

Since immune parameters of chickens are related to the genetic background [15,16,17] and to differences in phytate degradation [18,19], an interplay between dietary P and Ca supply and strain-specific factors on the immune system in chickens can be expected. Respective knowledge in laying hens is rarely available. So far, no study has addressed the effects of varying dietary P and Ca on the number and distribution of leukocyte subsets of both the systemic and gut-associated immune system, and the functionality of lymphocytes.

Therefore, the objective of this study was to evaluate the impact of the interplay of dietary P and Ca on the peripheral and gut-associated immune system in two laying hen strains. We hypothesized that variations in dietary P and Ca might affect the immune system by modifying the distribution and function of immune cells, and that these differences are further modulated by genetic background.

## 2. Materials and Methods

### 2.1. Birds and Housing

The study was conducted according to the ethical and animal care guidelines of the German Animal Welfare Legislation and approved by the local authority Animal Ethics Committee (Regional Council Tübingen, approval number HOH 50/17 TE). Animals were kept at the farm animal research center of the University of Hohenheim (Agricultural Experimental Station, Unterer Lindenhof, Eningen, Germany).

The research presented here complements and extends a recent publication [10]. The 2 × 2 × 2 factorial experiment includes the factors strain, dietary P, and dietary Ca concentration. To represent substantial degrees of genetic differentiation, hatchlings of a brown (Lohmann Brown-Classic, LB) and a white (Lohmann LSL-Classic, LSL) layer strain were obtained from Lohmann Tierzucht GmbH (Cuxhaven, Germany). In total, 158 LB and 144 LSL female hatchlings of 16 nonrelated roosters for each strain were raised together in one group in a deep-litter pen and received the same corn-soybean meal-based diets prior to the experimental phase, according to their age and the recommendations of the breeding company. For the experimental phase, 10 roosters per strain were chosen based on the average body weight of their offspring and four LSL and LB hens of each of the 10 roosters per strain were randomly selected, resulting in 40 selected hens per strain.

At 27 weeks of age, hens were placed individually in metabolic units (1 m × 1 m × 1 m) that were distributed in the barn in three windowless rooms, which were connected with each other by a door, in a completely randomized block design. Cages were equipped with a wooden perch, a nest, a feeding trough, water cups, and a wire mesh floor. Each hen had visual contact to another hen. Artificial lighting of 16 h of light and 8 h of darkness was provided during the experimental period, and the barn temperature was set to 18 °C. The hens were given one of four experimental diets that differed in P and Ca concentration, evenly distributed across the strain. This arrangement resulted in 20 replicates per dietary treatment and in 10 replicates per strain and dietary treatment.

### 2.2. Diets

The experimental diets were based on corn and soybean meal and were formulated to meet the nutrient requirement according to the recommendations of the German Society for Nutrition Physiology (Gesellschaft für Ernährungsphysiologie, GfE) [20], with the exception of P and Ca in the respective treatments [10]. Diets were supplemented with either standard (5.3 g/kg dry matter (DM); P+) or reduced (4.7 g/kg DM; P−) P concentration and with either standard (39.6 g/kg dry matter; Ca+) or reduced (33.9 g/kg DM; Ca−) Ca concentration, resulting in four different diets (P+ Ca+, P+ Ca−, P− Ca+, P− Ca−). Reduced concentrations amounted to 80% and 85% of hens’ actual P and Ca requirement, respectively. Diets were provided to the hens upon their placement in the metabolic units in wk 27 until week 31. Birds had free access to feed and water throughout the experiment.

### 2.3. Sampling and Sample Preparation

Blood samples of all hens were taken in wk 30 by vena ulnaris puncture within 2 min of the hen being removed from the metabolism unit in order to avoid an acute stress response to capture and handling. Blood samples were collected into 2 mL Eppendorf tubes and 5 mg/mL EDTA, and were fixed by the addition of TransFix^®^ reagent (Caltag Medsystems Ltd., Buckingham, UK) according to the manufacturer’s instructions for flow cytometric analysis. Unfixed blood samples for antibody analyses were centrifuged (15 min at 2000× *g* and 4 °C) and stored at −20 °C until measurement.

Immunological assessments also included bile, the spleen as a major secondary lymphatic organ [21], and the cecal tonsils as major lymphoid tissues within the gut [22]. Tissue and bile sampling was conducted in week 31 on four consecutive days with treatments equally distributed among the days. Hens were individually sedated with a gas mixture of 35% CO_2_, 35% N_2_, and 30% O_2_, and killed by decapitation. Spleen and cecal tonsils were taken and stored for transport on ice in PBS with 1% Fetal Bovine Serum (FBS) and 50 µg/mL gentamycin. Bile was withdrawn by puncture of the gall bladder and frozen at −20 °C until analysis of immunoglobulin (Ig) A concentrations by ELISA technique. Lymphatic tissue was processed according to Hofmann and Schmucker [23]. In brief, the spleen was cut into pieces under sterile conditions and dissociated with a gentleMACS Dissociator (Miltenyi Biotec, Bergisch Gladbach, Germany). Accrued cell suspension was then applied to a 40 µm MACS SmartStrainer (Miltenyi Biotec). Flowthrough was centrifuged and the cell pellet resuspended in PBS with 1% FBS. Intraepithelial lymphocytes of one randomly chosen cecal tonsil were removed from the mucosa by shaking the tissue in Hanks’ Balanced Salt solution (without Mg^2+^ and Ca^2+^) supplemented with 5 mM EDTA, 5% FBS, and 1 mM Dithiothreitol (Sigma Aldrich, St. Louis, MO, USA). Samples were put onto a 40 µm MACS SmartStrainer (Miltenyi Biotec), with the flowthrough containing desired intraepithelial lymphocytes. After two washing steps, the single-cell suspension was centrifuged and the cell pellet resuspended in PBS with 1% FBS. The final volume of spleen and intraepithelial lymphocytes suspension was determined and stored on ice until further processing.

### 2.4. Flow Cytometric Analysis

For discrimination and counting of various leukocyte types, stabilized whole blood samples as well as single-cell suspensions of spleen and intraepithelial lymphocytes of cecal tonsils were stained with fluorescently labeled antibodies and analyzed by flow cytometry according to Hofmann and Schmucker [23]. Analyses were performed on a BD FACSCanto™ II (BD Biosciences, Heidelberg, Germany) equipped with a 488 nm blue laser, 630 nm red laser, and a 405 nm violet laser, and by using BD FACSDiva™ Software II (BD Biosciences). At least 10,000 CD45^+^ cell events per blood sample and 50,000 CD45^+^ cell events per tissue sample were analyzed. The following chicken-specific fluorochrome-labeled antibodies against respective cell surface markers were used: CD45 (clone LT40; #8270-11), Monocyte/Macrophage (clone Kul01; #8420-09), CD4 (clone CT-4; #8210-26 and #8210-09), CD8α (clone CT-8; #8220-02), Bu-1 (clone AV20; #8395-02) (all SouthernBiotech, Birmingham, AL, USA), CD41/61 (clone 11C3; #MCA2240GA; BioRad, Santa Rosa, CA, USA), and TCRγδ (clone TCR1; #NBP1-28275PCP; Novus Biologicals, Centennial, CO, USA). A mouse IgM isotype control (clone 11E1; #0101-11; SouthernBiotech) was used to exclude nonspecific binding of anti-CD45 to verify correct numbers of total leukocytes. Cells of blood, spleen, and cecal tonsil were stained by the respective antibody combination for 45 min in the dark at room temperature. Dead cells in cell suspensions of spleen and cecal tonsil were excluded by SYTOX^®^ Blue Dead Cell Stain (#S34857; ThermoFisher Scientific, Waltham, MA, USA). Specific immune cells were classified by the combination of surface marker expression as follows: total leukocytes (CD45^+^), thrombocytes (CD45^dim^/CD41/61^+^) (only blood and spleen), monocytes (CD45^+^/Kul01^+^) (only blood and spleen), CD4^+^ T cells (CD45^+^/CD4^+^/TCRγδ^−^), CD8α^+^ T cells (CD45^+^/CD4^−^/TCRγδ^−^/CD8α^+^), γδ T cells (CD45^+^/Kul01^−^/CD4^−^/TCRγδ^+^), and B cells (CD45^+^/Kul01^−^/Bu-1^+^). Heterophils in blood were identified based on their FSC/SSC characteristics. Absolute numbers of leukocytes per µL blood or tissue were determined using BD Trucount^™^ tubes (#340334; BD Biosciences) according to the manufacturer’s instructions. Absolute cell numbers of leukocyte subsets were calculated by combining cell frequencies with total leukocyte counts. Total T cells were calculated by the addition of CD4^+^ T cells, CD8α^+^ T cells, and γδ T cells.

### 2.5. Isolation of Splenic Mononuclear Cells

Splenic mononuclear cells were separated from the cell suspension by density gradient (Biocoll, 1.077 g/mL; Biochrom, Berlin, Germany) centrifugation for 12 min at 600× *g* and 20 °C. After separation of the interphase, mononuclear cells were washed in PBS with 1% FBS and the cell pellet was resuspended in RPMI 1640 (Biochrom) with 10% FBS. Cell numbers were determined using a Z2 Coulter Counter (Beckman Coulter, Krefeld, Germany).

### 2.6. Splenic Lymphocyte Proliferation Assay

Quantification of splenocyte proliferation capacity was examined in vitro by a mitogen-induced lymphocyte transformation test using ^3^H-thymidine incorporation. One hundred fifty thousand cells per well were transferred into 96-well round-bottom cell culture plates (Neolab, Heidelberg, Germany) and stimulated in triplicates per treatment with either 10 μg/mL concanavalin A (ConA), pokeweed mitogen (PWM) (both Sigma Aldrich), or left without stimulation as negative control. Cells were incubated at 41 °C and 5% CO_2_ for 48 h. Afterward, 0.25 μCi ^3^H-thymidine (PerkinElmer, Rodgau, Germany) per well was added for an additional 24 h. Cells were harvested on glass fiber filters (Skatron, Lier, Norway) and the amount of radioactivity incorporated was evaluated in cpm by a liquid scintillation analyzer (PerkinElmer). Mean of cpm for each triplicate was calculated and delta cpm (delta cpm = stimulated cells−unstimulated cells) for ConA and PWM was generated. Intra-assay coefficient of variation (CV) for delta cpm of ConA was 11% and for delta cpm of PWM 15%. To correct for variation between the single assays, data were normalized against a control.

### 2.7. Stimulation of IFN-γ Production

For the determination of mitogen-induced IFN-γ production of splenocytes, 5 × 10^5^ mononuclear cells per well were transferred to 96-well flat-bottom cell culture plates (Neolab). Cells were stimulated in duplicate with either 10 μg/mL ConA, 10 μg/mL PWM (both Sigma Aldrich), or left without stimulation as negative control. After 25 h at 41 °C and 5% CO_2_ incubation, supernatants were collected and stored at −20 °C until measurement.

### 2.8. Enzyme-Linked Immunosorbent Assay

*Antibody concentration:* Concentrations of IgY and IgM in plasma and IgA in plasma and bile were measured by ELISA at room temperature. The 96-well flat-bottom microtiter plates (ThermoFisher Scientific) were coated with 200 ng/well of either goat anti-chicken IgY Fc antibody (#A30-104-A), goat anti-chicken IgM antibody (#A30-102-A) or goat anti-chicken IgA antibody (#A30-103-A) (all Bethyl Laboratories, Montgomery, TX, USA), diluted in coating buffer (15 mM NaHCO_3_, pH 9.6), and incubated overnight at 4 °C. After being diluted in buffer (50 mM Tris, 0.0027 M KCL, 0.14 M NaCL; pH 8.0), plasma (IgY 1:200,000; IgM 1:10,000; IgA 1:2000) and bile samples (IgA: 1:1,000,000) were added to the plates after blocking with BSA for 30 min (Roth, Karlsruhe, Germany). Bound anti-IgY, anti-IgM, and anti-IgA antibodies in plasma and bile samples were detected with 100 ng/well of either horseradish peroxidase-labeled goat anti-chicken IgY Fc (#A30-104-P), goat anti-chicken IgM (#A30-102-P), or goat anti-chicken IgA antibody (#A30-103-P) (all Bethyl Laboratories) diluted 1:100,000 in coating buffer, respectively. After 1 h of incubation at room temperature, tetramethylbenzidine (AppliChem, Darmstadt, Germany) was added and color formation was stopped after 20 min with 2 M H_2_SO_4_ (Roth, Karlsruhe, Germany). Plates were washed five times between each step with washing buffer (50 mM Tris, 0.0027 M KCL, 0.14 M NaCl, 0.05% Tween 20; pH 8.0). All samples were quantified by reference to a calibration curve set up with a pooled plasma control whose IgY, IgM, and IgA concentration was determined in advance with Chicken IgG ELISA Kit (#E33-104), Chicken IgM ELISA Kit (#E33-102) and Chicken IgA ELISA Kit (#E33-103), all from Bethyl Laboratories (Montgomery, TX, USA). The calibration curve of IgY, IgM, and IgA ranged from 4.96 to 300 ng/mL, from 7.8 to 500 ng/mL, and from 15.63 to 1000 ng/mL, respectively. The absorbance was measured at 450 nm and antibody concentration was calculated relative to the absorbance of the calibration curve. CV of intra-assay in plasma was 4.3% for IgY, 7.6% for IgM and 3.7% for IgA, and 4.3% for IgA in bile. CV of inter-assay in plasma was 5.7% for IgY, 7.0% for IgM and 2.9% for IgA, and 7.3% for IgA in bile.

*IFN-γ Concentration:* Cell culture supernatants, which were stimulated in duplicate either with ConA or PWM or not stimulated (medium only), were pooled. IFN-γ concentration was analyzed in triplicates using a chicken IFN-γ CytoSet™ ELISA kit (#CAC1233; Invitrogen™, ThermoFisher Scientific), following manufacturer’s instructions. The calibration curve was derived from serial dilutions of a standard given in the kit, and IFN-γ concentration was calculated relative to the absorbance of the calibration curve. The coefficient of the intra-assay variation was 3.9% for a pooled ConA and PWM stimulated sample. To compensate for the variability during stimulation process and ELISA assay, data were normalized against a control sample.

### 2.9. Statistical Analysis

Statistical analysis was performed using a linear mixed model with the PROC MIXED procedure of the software package SAS (version 9.4; SAS Institute Inc., Cary, NC, USA). Residuals were checked for normal distribution and homogeneous error via graphical check of residual plots [24]. In order to fulfill model assumptions, all variables had to be logarithmized. The individual hen was considered as the experimental unit. The following model was used:

Y_ijklmn_ = μ + α_i_ + β_j_ + γ_k_ + (αβ)_ij_ + (αγ)_ik_ + (βγ)_jk_ + (αβγ)_ijk_ + δ_l_ + φ_m_ + χ_n_ + ε_ijklmn_

where Y_ijklmn_ = response variable; μ = overall mean; α_i_ = effect of strain (fixed); β_j_ = effect of dietary P (fixed); γ_k_ = effect of dietary Ca (fixed); (αβ)_ij_ = interaction of strain and dietary P (fixed); (αγ)_ik_ = interaction of strain and dietary Ca (fixed); (βγ)_jk_ = interaction of dietary P and dietary Ca (fixed); (αβγ)_ijk_ = interaction of strain; dietary P and dietary Ca (fixed); δ_l_ = room (fixed); φ_m_ = block (random), which includes the different sampling days; χ_n_ = father/rooster (random); and ε_ijklmn_ = residual error.

In order to eliminate duration of blood sampling from the respective parameters, data were statistically corrected by including sampling time for blood as a covariate. Covariables were checked for significance and were dropped from the model if they were not significant. In the case of significance of F-tests, a Fisher’s LSD test was used for multiple pairwise post hoc testing. Results are presented as LSmeans and pooled SEM of the back-transformed data. Statistical significance was declared at *p* < 0.05.

## 3. Results

### 3.1. Impact of Strain, Dietary P and Ca on Number and Distribution of Immune Cells

#### 3.1.1. Blood

The results of the impact of strain, dietary P and Ca on the number of immune cells in whole blood are presented in Table 1. The interaction of strain, dietary P and Ca considerably affected the number of γδ T cells (*p* = 0.032 for strain × P × Ca). In LB hens, but not in LSL hens, the number of total γδ T cells was lower when fed P−Ca− than when fed P+Ca− (post hoc testing, *p* = 0.036) or P−Ca+ (post hoc testing, *p* = 0.008). Dietary P affected monocytes dependent on strain (*p* = 0.006 for strain × P) to the effect that numbers in LB hens, but not in LSL hens, were higher when fed the P− diet (post hoc testing, *p* = 0.026). Other cell types were not affected by any interaction of strain, dietary P, and dietary Ca (*p* > 0.05). Irrespective of strain, P and Ca affected the number of monocytes (*p* = 0.039 for P × Ca). Hens fed P−Ca− compared to P−Ca+ had higher numbers of monocytes (post hoc testing, *p* = 0.012). Dietary Ca affected the number of total T cells (*p* = 0.036 for Ca) and CD4^+^ T cells (*p* = 0.042 for Ca). Hens fed the Ca− diets had lower numbers of total T cells and CD4^+^ T cells than hens fed the Ca+ diets. Dietary P influenced the number of B cells (*p* = 0.049 for P). Hens fed the P− diets had higher numbers of B cells compared to hens fed the P+ diets. The number of total leukocytes (*p* = 0.041 for strain), total T cells (*p* < 0.001 for strain), CD4^+^ T cells (*p* = 0.022 for strain), CD8α^+^ T cells (*p* < 0.001 for strain), and B cells (*p* = 0.011 for strain) were higher in LSL than in LB hens, and the number of thrombocytes (*p* < 0.001 for strain) and heterophils (*p* < 0.001 for strain) were lower in LSL hens than in LB hens.

#### 3.1.2. Spleen

Results of the impact of strain, dietary P and Ca on the number of immune cells in the spleen are presented in Table 2. The number of immune cells was not affected by any interactions of strain, dietary P and Ca, nor by dietary P and Ca as single main effects (*p* > 0.05). However, LSL hens had higher average numbers of leukocytes (*p* = 0.015 for strain), thrombocytes (*p* = 0.010 for strain), monocytes (*p* = 0.015 for strain), T cells (*p* < 0.001 for strain), T helper cells (*p* < 0.001 for strain), and cytotoxic T cells (*p* < 0.001 for strain) than LB hens.

#### 3.1.3. Cecal Tonsils

Results of the impact of strain, dietary P and Ca on the number of immune cells among intraepithelial lymphocytes of the cecal tonsils are presented in Table 3. The numbers of immune cells were not affected by the interaction of strain, dietary P and Ca, nor by the interaction of strain and dietary Ca or the interaction of dietary P and Ca (*p* > 0.05). However, the interaction of strain and dietary P had an impact on the number of γδ T cells (*p* = 0.039 for strain × P) to the effect that LB hens, but not LSL hens, had higher numbers when fed the P− diets compared to the P+ diets (post hoc testing, *p* = 0.038). Irrespective of strain, dietary Ca had an impact on the number of total leukocytes (*p* = 0.020 for Ca), total T cells (*p* = 0.004 for Ca), γδ T cells (*p* = 0.027 for Ca), and CD8α^+^ T cells (*p* = 0.005 for Ca), and tended to affect the number of CD4^+^ T cells (*p* = 0.054 for Ca). Hens fed the Ca− diets had lower numbers of each cell type than hens fed the Ca+ diets. LSL hens had higher numbers of total leukocytes (*p* = 0.009 for strain) and B cells (*p* = 0.001 for strain) than LB hens.

### 3.2. Impact of Strain, Dietary P and Ca on Functionality of Immune Cells

#### 3.2.1. Antibody Concentrations

Results of the impact of strain, dietary P and Ca on antibody concentrations in plasma and bile are presented in Table 4. The interaction of strain, dietary P and Ca and the interaction of strain and dietary Ca had no influence on antibody concentrations in plasma and bile (*p* > 0.05). P affected IgA levels in plasma dependent on strain (*p* = 0.023 for strain × P). LSL hens, but not LB hens, had higher levels of IgA in plasma when fed the P− diets compared to the P+ diets (post hoc testing, *p* = 0.038). IgA concentrations in bile were affected by dietary P (*p* = 0.049 for P) and Ca (*p* = 0.007 for Ca). Hens fed the P− or Ca− diets had higher concentrations of IgA in bile compared to hens fed P+ or Ca+ diets. IgY and IgM concentrations in plasma did not differ among treatments (*p* > 0.05).

#### 3.2.2. Lymphocyte Proliferation Capacity and IFN-γ Concentration

Results of the impact of strain, dietary P and Ca on lymphocyte proliferation and IFN-γ production of splenocytes are presented in Table 5. Lymphocyte proliferation capacity was only affected by strain. Splenic lymphocytes of LSL hens showed a greater proliferation response to mitogens ConA and PWM than the splenic lymphocytes of LB hens (both *p* < 0.001 for strain). Although not significant, proliferation to ConA in hens fed the P− diets tended to be higher compared to hens fed the P+ diets (*p* = 0.090 for P). IFN-γ concentrations after stimulation with PWM was affected by strain and dietary P (*p* = 0.003 for strain × P). In LB hens, but not LSL hens, mean IFN-γ concentrations were greater when hens were fed P− diets (post hoc testing, *p* = 0.013). There was no effect of dietary P and Ca on IFN-γ concentrations after stimulation with ConA (*p* > 0.05). However, IFN-γ production in response to ConA (*p* = 0.001 for strain) was higher in LSL hens than in LB hens.

## 4. Discussion

The present study showed that reductions in dietary P and Ca are associated with immunomodulatory effects on the peripheral and gut-associated immune system in laying hens. In general, immune cell numbers as well as the mitogen-induced response of innate and adaptive immune cells were increased in hens fed the P− diets, while hens on a Ca− diet had reduced numbers of cells of the adaptive immune system. The genetic background appears to influence the impact of dietary P, but not of dietary Ca.

LB hens were apparently more influenced by low dietary P, as they showed an increased number of peripheral monocytes and γδ T cells in the cecal tonsils as well as higher in vitro IFN-γ production of splenocytes, whereas these parameters were unaffected in LSL hens when fed the P− diets. Irrespective of the strain, diets low in P showed a stimulating effect on circulating B cells and IgA concentrations in bile. Additional effects were observed when both P and Ca were low. A P−Ca− diet increased the number of circulating monocytes in both strains, while the number of γδ T cells was only decreased in LB hens. Generally, immune parameters were higher in hens fed a P− diet compared to a P+ diet, suggesting an improved immune function. Furthermore, the immunomodulatory effects were seen more often in LB hens than in LSL hens. These results indicate, with respect to the immune system, that different strains of laying hens react differently to the same feed composition.

At first sight, the results of the present study may appear contradictory to other studies in which higher P availability was associated with enhanced immune function [13,14,25,26,27,28]. However, in these studies, experimental groups encompassed diets with higher P availability compared to standard diets with recommended levels of P. These higher P levels were achieved by either supplementation with high levels of nonphytate-P or by addition of exogenous phytase. This contrasts with the study design of the present study where hens in the experimental groups were fed diets supplemented with lower mineral P than recommended compared to the standard control group receiving P in recommended amounts. Thus, in order to enhance P availability, hens of the present study had to hydrolyze phytate by endogenous mucosal and microbial phytases and phosphatases.

Low mineral P diets were shown to increase intestinal phytase activity in chickens and promote the substantial degradation of phytate-P, and therefore the absorption of P [29,30,31,32]. Lower inositol phosphates and *myo*-inositol, as a result of InsP_6_ degradation, are also associated with various effector functions in immune cells, including proliferation, cytokine production, and cytotoxicity [33,34], by acting as a second messenger [35,36]. In the companion project, Sommerfeld et al. [10] did not find differences in lower inositol phosphates, but a tendency (*p* = 0.088) towards higher concentrations of *myo*-inositol in the ceca of hens fed the P− diet. Moreover, Sommerfeld et al. [19] suggested strain differences in digestive phytate degradation and/or *myo*-inositol uptake through the intestinal wall. LB hens showed higher mucosal phosphatase and phytase activity, higher concentrations of *myo*-inositol and InsP_x_ in the digestive tract, and higher concentrations of *myo*-inositol in blood and egg compared to LSL hens. This is consistent with our observations regarding the measured immune traits, as LB hens were shown to react more sensitively than LSL hens to some immune parameters. Nevertheless, Sommerfeld et al. [10] also described different mechanisms of LSL and LB hens with regard to P absorption to meet their respective P requirements. This might explain strain-dependent impacts of P on immune systems with higher cell numbers in LB hens, but higher IgA levels in LSL hens fed P− diets. However, further research is needed to resolve underlying mechanisms of these strain-related immune-modulatory effects of dietary P. In general, the relationship between *myo*-inositol, InsP_x,_ and the immune response in poultry, particularly regarding dietary effects, has not been the subject of research so far and needs further investigation to support this assumption

Another possible explanation for the immunomodulating properties of dietary P relates to microbial degradation process. Nutrients are very well known to modulate the gut microbiome in terms of diversity and composition which, in turn, has an impact on chicken health [37]. Bacterial fermentation products such as butyrate act as potent mediators of immune regulation in chickens [38,39]. High P availability was associated with an increase of butyrate-producing bacteria in cecal digesta, as shown in broiler chickens [40] and pigs [41]. Thus, it is also possible that dietary P modulates the immune system indirectly via the composition of the intestinal microbiome. In addition, strain-dependent divergences in microbiota between LB and LSL hens could explain the differential outcome of dietary intervention on the immune system found in the present study. In the future, it is expected that microbiota and network analysis in ongoing companion projects might help to resolve this interesting interaction.

Dietary Ca was also shown to be associated with alterations in several immune parameters in the present study. Irrespective of strain, hens fed the Ca− diets had lower numbers of immune cells, especially T cells and -subsets, in blood and cecal tonsils. The only enhanced parameter was a higher IgA concentration in bile. Ca deficiency leads to an increase in parathyroid hormone which, in turn, promotes the production of 1,25-dihydroxyvitamin D_3_ (1,25-(OH)_2_D_3_) [42]. 1,25(OH)_2_D_3_ interacts with the vitamin D receptors that have been found in various cells in the body, including immune cells [43], indicating immunomodulatory properties [44]. In vitro studies with chickens have demonstrated that 1,25(OH)_2_D_3_ suppresses T cell proliferation and T cell stimulatory function of antigen-presenting cells [45,46], and improves antibacterial defense mechanisms of monocytes/macrophages by enhancing the production of nitric oxide [46] and antimicrobial peptides like β-defensins [47]. The functionality of innate immune cells was not tested in the present study, but the number of monocytes in blood was higher in hens fed the P−Ca− diet, which might be an indication of improved innate immune function.

The results of the present study suggest immunomodulatory properties of dietary P and Ca on the peripheral and gut-associated immune system in laying hens. The results so far suggest that a monocalcium phosphate supplementation lower than the current recommendation would be beneficial with respect to immune function. In contrast, lower Ca concentrations had negative effects on adaptive cellular immunity.

Obviously, further research is needed to understand the relationship between dietary P and Ca and the immune system in more detail. Not only deficiency, but also excessive supply can decrease mineral digestibility and absorption [48,49] and therefore may cause immunosuppression in chickens [27]. Especially, the theory that hens fed the P− diets have a higher concentration of available P for the immune system needs further investigation. We assume that diets without any mineral P supplementation will fully exploit the ability of the intestine to perform endogenous phytase activity and hydrolyze phytate by forming lower inositol phosphates and *myo*-inositol with a possible beneficial role for immunomodulation.

In the present study, marked differences between LSL and LB hens in almost all immune parameters were found, thereby confirming a substantial genetic impact for immune traits in laying hens [15,50]. LSL hens were found to have more lymphocytes than LB hens in blood, and partially also in the spleen and the cecal tonsils. They also showed higher mitogen-induced proliferation and IFN-γ production capacity of splenic lymphocytes. In contrast, LB hens had higher numbers of thrombocytes and heterophils as well as higher concentrations of IgA in blood. If and how these differences may influence overall chicken health and protection against infection needs to be evaluated in further studies, but observations might point to a bias of the adaptive immune response in these two hen strains, with pronounced cellular responses in LSL and stronger humoral responses in LB hens.

## 5. Conclusions

In conclusion, the results of the present study demonstrated several enhancing effects of the P− diets on the number of innate and adaptive immune cells as well as on antibody concentrations in both strains, but especially in LB hens. There are several possible explanations for the immunomodulating properties of dietary P, such as the presence of *myo*-inositol or the composition of the microbiome in the gut. Nevertheless, further studies are needed to elucidate the impact of dietary mineral formulation on InsP_x_ degradation and microbiota composition, and their potential to modulate the immune system for better or worse. Based on the current results, a reduction of dietary Ca is not recommended as the numbers of the various T cell subtypes is decreased, which could lead to impaired cellular adaptive immune responses.

## Figures and Tables

**Table 1:** Effect of strain, dietary P and Ca on the number of immune cells (#/µL) in whole blood.

Strain	Dietary P	Dietary Ca	Leukocytes	Thrombocytes	Monocytes	Heterophils	T Cells	CD4^+^ T Cells	γδ T Cells	CD8α^+^ T Cells	B Cells
LSL ^1^	P+	Ca+	35,807	54,864	2677	3720	14,924	7036	4612	3144	1926
LSL	P+	Ca−	32,784	50,050	2627	3410	13,062	5892	4077	2827	2045
LSL	P−	Ca+	34,513	51,142	2015	3699	13,837	6705	4293	2745	2354
LSL	P−	Ca−	33,428	51,913	2428	3011	13,194	6147	3968	2998	2522
LB ^2^	P+	Ca+	31,919	64,562	1623	7345	9974	5843	2241	1648	1322
LB	P+	Ca−	29,541	69,667	1541	6459	9346	5095	2701	1343	1398
LB	P−	Ca+	33,210	64,732	1674	7445	10,484	5436	2942	1947	1776
LB	P−	Ca−	31,722	70,014	2368	7310	9550	5656	1995	1636	1616
	SEM		1539	2615	208	637	731	393	365	258	287
	*p*-values	Strain × P × Ca	0.847	0.413	0.501	0.486	0.473	0.620	0.032	0.612	0.708
									*LB: P−Ca− < P+Ca−* *P−Ca− < P−Ca+*		
		Strain × P	0.319	0.745	0.006	0.416	0.389	0.827	0.810	0.174	0.946
					*LB: P− > P+*						
		Strain × Ca	0.981	0.073	0.655	0.656	0.887	0.340	1.000	0.265	0.693
		P × Ca	0.494	0.392	0.039	0.979	0.717	0.129	0.065	0.486	0.740
					*P−Ca− > P−Ca+*						
		P	0.466	0.837	0.732	0.982	0.971	0.879	0.646	0.375	0.049
											*P− > P+*
		Ca	0.062	0.542	0.111	0.183	0.036	0.042	0.156	0.221	0.832
							*Ca− < Ca+*	*Ca− < Ca+*			
		Strain	0.041	<0.001	0.001	<0.001	<0.001	0.022	<0.001	<0.001	0.011
			*LSL > LB*	*LSL < LB*	*LSL > LB*	*LSL < LB*	*LSL > LB*	*LSL > LB*	*LSL > LB*	*LSL > LB*	*LSL > LB*

Data are given as LSmeans and pooled SEM; *n* = 10. ^1^ LSL = Lohmann LSL-Classic. ^2^ LB = Lohmann Brown-Classic. In case of significance of any fixed effect (*p* < 0.05), significant differences of the post hoc test (Fishers LSD test; *p* < 0.05) and direction of effects are stated below.

**Table 2:** Effect of strain, dietary P and Ca on the number of immune cells (×10^6^) in the spleen.

Strain	Dietary P	Dietary Ca	Leukocytes	Thrombocytes	Monocytes	T Cells	CD4^+^ T Cells	γδ T Cells	CD8α^+^ T Cells	B Cells
LSL ^1^	P+	Ca+	2741	241	6.25	1952	543	453	949	594
LSL	P+	Ca−	2866	226	5.72	1989	554	444	977	701
LSL	P−	Ca+	2730	242	6.88	1860	542	435	874	680
LSL	P−	Ca−	2204	183	4.89	1485	406	339	734	575
LB ^2^	P+	Ca+	1965	167	3.29	1162	286	356	450	577
LB	P+	Ca−	2157	183	3.76	1324	298	388	611	616
LB	P−	Ca+	2136	180	4.37	1237	346	346	531	685
LB	P−	Ca−	2219	173	4.55	1282	315	398	555	699
	SEM		233	23.8	1.13	156	44.6	41.4	95.5	78.9
	*p*-values	Strain × P × Ca	0.475	0.805	0.757	0.604	0.560	0.362	0.875	0.396
		Strain × P	0.187	0.532	0.303	0.201	0.067	0.327	0.239	0.287
		Strain × Ca	0.291	0.259	0.245	0.199	0.470	0.113	0.185	0.803
		P × Ca	0.272	0.329	0.502	0.244	0.144	0.570	0.209	0.269
		P	0.585	0.595	0.427	0.283	0.827	0.315	0.416	0.487
		Ca	0.893	0.411	0.619	0.891	0.285	0.885	0.582	0.804
		Strain	0.015	0.010	0.015	<0.001	<0.001	0.189	<0.001	0.922
			*LSL > LB*	*LSL > LB*	*LSL > LB*	*LSL > LB*	*LSL > LB*		*LSL > LB*	

Data are given as LSmeans and pooled SEM; *n* = 10. ^1^ LSL = Lohmann LSL-Classic. ^2^ LB = Lohmann Brown-Classic. In case of significance of any fixed effect (*p* < 0.05), significant differences of the post hoc test (Fishers LSD test; *p* < 0.05) and direction of effects are stated below.

**Table 3:** Effect of strain, dietary P and Ca on the number of immune cells (×10^5^) amongst intraepithelial lymphocytes of the cecal tonsils.

Strain	Dietary P	Dietary Ca	Leukocytes	T Cells	CD4^+^ T Cells	γδ T Cells	CD8α^+^ T Cells	B Cells
LSL ^1^	P+	Ca+	651	406	72.6	92.4	232	142
LSL	P+	Ca−	616	373	68.5	76.0	225	164
LSL	P−	Ca+	654	388	83.8	85.7	214	173
LSL	P−	Ca−	523	309	58.0	68.2	178	140
LB ^2^	P+	Ca+	484	336	55.9	58.5	215	87.7
LB	P+	Ca−	452	299	55.2	61.7	176	96.8
LB	P−	Ca+	557	392	73.1	87.8	226	93.2
LB	P−	Ca−	396	265	50.2	64.4	142	64.6
	SEM		55.8	33.6	9.88	8.11	20.8	22.2
	*p*-values	Strain × P × Ca	0.720	0.640	0.897	0.272	0.724	0.821
		Strain × P	0.563	0.332	0.636	0.039	0.634	0.431
						*LB: P− > P+*		
		Strain × Ca	0.653	0.471	0.923	0.577	0.144	0.682
		P × Ca	0.129	0.132	0.108	0.192	0.172	0.101
		P	0.604	0.474	0.716	0.374	0.122	0.546
		Ca	0.020	0.004	0.054	0.027	0.005	0.497
			*Ca− < Ca+*	*Ca− < Ca+*		*Ca− < Ca+*	*Ca− < Ca+*	
		Strain	0.009	0.108	0.169	0.086	0.142	0.001
			*LSL > LB*					*LSL > LB*

Data are given as LSmeans and pooled SEM; *n* = 10. ^1^ LSL = Lohmann LSL-Classic. ^2^ LB = Lohmann Brown-Classic. In case of significance of any fixed effect (*p* < 0.05), significant differences of the post hoc test (Fishers LSD test; *p* < 0.05) and direction of effects are stated below.

**Table 4:** Effect of strain, dietary P, and dietary Ca on antibody concentrations in plasma and bile.

				Plasma		Bile
Strain	Dietary P	Dietary Ca	IgY [mg/mL]	IgM [µg/mL]	IgA [µg/mL]	IgA [mg/mL]
LSL ^1^	P+	Ca+	15.4	777	186	37.6
LSL	P+	Ca−	12.4	789	233	55.0
LSL	P−	Ca+	9.0	780	335	62.2
LSL	P−	Ca−	12.7	713	292	59.6
LB ^2^	P+	Ca+	13.8	715	453	38.5
LB	P+	Ca−	14.6	606	378	48.1
LB	P−	Ca+	12.4	664	351	37.1
LB	P−	Ca−	12.7	777	388	55.9
	SEM		2.19	69.2	54.9	6.10
	*p*-values	Strain × P × Ca	0.232	0.088	0.157	0.085
		Strain × P	0.596	0.271	0.023	0.181
					*LSL: P− > P+*	
		Strain × Ca	0.944	0.794	0.708	0.396
		P × Ca	0.296	0.378	0.854	0.499
		P	0.138	0.754	0.193	0.049
						*P− > P+*
		Ca	0.684	0.737	0.993	0.007
						*Ca− > Ca+*
		Strain	0.456	0.253	0.008	0.113
					*LSL < LB*	

Data are given as LSmeans and pooled SEM; *n* = 9–10. ^1^ LSL = Lohmann LSL-Classic. ^2^ LB = Lohmann Brown-Classic. In case of significance of any fixed effect (*p* < 0.05), significant differences of the post hoc test (Fishers LSD test; *p* < 0.05) and direction of effects are stated below.

**Table 5:** Effect of strain, dietary P, and dietary Ca on lymphocyte proliferation capacity and IFN-γ concentrations of splenocytes.

Strain	Dietary P	Dietary Ca	Lymphocyte Proliferation		IFN-γ	
			ConA [∆ cpm]	PWM [∆ cpm]	ConA [pg/mL]	PWM [pg/mL]
LSL ^1^	P+	Ca+	681	1401	3981	4438
LSL	P+	Ca−	1076	1784	3999	4882
LSL	P−	Ca+	1044	1761	4700	2455
LSL	P−	Ca−	1120	1668	5555	3515
LB ^2^	P+	Ca+	144	929	500	1239
LB	P+	Ca−	186	814	695	1603
LB	P−	Ca+	332	996	1925	2489
LB	P−	Ca−	228	951	1056	2829
	SEM		211	194	1831	955
	*p*-values	Strain × P × Ca	0.783	0.297	0.493	0.574
		Strain × P	0.514	0.863	0.424	0.003
						*LB: P− > P+*
		Strain × Ca	0.460	0.317	0.779	0.922
		P × Ca	0.251	0.565	0.630	0.848
		P	0.090	0.291	0.158	0.621
		Ca	0.646	0.980	0.949	0.232
		Strain	<0.001	<0.001	0.001	0.014
			*LSL > LB*	*LSL > LB*	*LSL > LB*	*LSL > LB*

Data are given as LSmeans and pooled SEM; *n* = 10. ^1^ LSL = Lohmann LSL-Classic. ^2^ LB = Lohmann Brown-Classic. In case of significance of any fixed effect (*p* < 0.05), significance differences of the post hoc test (Fishers LSD test; *p* < 0.05) and direction of effects are stated below.

## Data Availability

The data presented in this study are available in the present article.
